# Non-alcoholic fatty pancreas disease (NAFPD) as a pre-neoplastic niche: Metabolic and inflammatory Gateways to pancreatic ductal adenocarcinoma^☆^

**DOI:** 10.1016/j.jcte.2025.100424

**Published:** 2025-11-06

**Authors:** Hope Onohuean, Ngozi F. Nnolum-Orji, Sarad Pawar Naik Bukke, Kasim Sakran Abass, Abdullateef Isiaka Alagbonsi, Yahya E. Choonara

**Affiliations:** a Wits Advanced Drug Delivery Platform Research Unit, Department of Pharmacy and Pharmacology, School of Therapeutic Sciences, Faculty of Health Sciences, University of the Witwatersrand, 7 York Road, Parktown, Johannesburg 2193, South Africa; b Biomolecules, Metagenomics, Endocrine and Tropical Disease Research Group (BMETDREG), Kampala International University, Western Campus, Ishaka-Bushenyi, Uganda; c Biopharmaceutics Unit, Department of Pharmacology and Toxicology, School of Pharmacy, Kampala International University, Uganda; d Department of Pharmaceutics and Pharmaceutical Technology, Kampala International University, Western Campus, P.O. Box 71, Ishaka-Bushenyi, Uganda; e Department of Physiology, Biochemistry, and Pharmacology, Biochemistry, and Pharmacology, College of Veterinary Medicine, University of Kirkuk, Kirkuk 36001, Iraq; f Department of Physiology, School of Medicine and Pharmacy, College of Medicine and Health Sciences, University of Rwanda, Huye, Rwanda; g Wits Infectious Diseases and Oncology Research Institute, Faculty of Health Sciences, University of the Witwatersrand, 7 York Road, Parktown, Johannesburg 2193, South Africa

**Keywords:** Non-alcoholic, Fatty pancreas disease, Pre-neoplastic, Metabolic, Inflammatory, Pancreatic ductal adenocarcinoma

## Abstract

Non-alcoholic fatty pancreas disease (NAFPD), marked by ectopic triglyceride accumulation in the exocrine pancreas, is increasingly observed yet its recognition as a cancer-predisposing condition remains limited. We synthesize evidence supporting NAFPD as an early and modifiable niche for pancreatic ductal adenocarcinoma (PDAC), using a PRISMA-ScR-guided framework. The findings were synthesized into three domains: epidemiological risk, metabolic–inflammatory signaling, and immune–stromal remodeling. Mechanisms include palmitate-induced ER stress, ROS-driven NLRP3–IL-1β and STAT5 signaling, and KRAS^G12D-mediated lipotoxicity. Lipid-laden stellate cells promote fibrosis, immunosuppression, and epithelial–mesenchymal transition. NAFPD may represent an early, modifiable PDAC niche, warranting further imaging–omic studies and targeted prevention trials.

## Introduction

Non-alcoholic fatty pancreas disease (NAFPD), marked by ectopic fat accumulation in the pancreas (pancreatic steatosis) without substantial alcohol consumption, is becoming acknowledged as a pathological condition separate from obesity or hepatic steatosis [1]. It was once thought to be a harmless illness, but it has since been linked to pancreatic and metabolic disorders and even cancer. Its prevalence has been reported as 16 % − 35 % among Asians [2] and 11 % among Chinese adults [3], while a *meta*-analysis from 11 studies yielded a prevalence of 33 % [4]. Risk factors, including obesity, age, and gender [3], have been associated with the onset of NAFPD.

The NAFPD is detected using various methods, including histology, abdominal ultrasonography, endoscopic ultrasound, computed tomography (CT), and magnetic resonance imaging (MRI). While the histological method is the gold standard that can accurately detect the pancreas fat content, it is hardly used practically due to the complex anatomical position of the pancreas, as this method requires a histological biopsy taken invasively [5]. The abdominal ultrasound is a convenient, affordable, and non-invasive method with a high penetration rate, which adopts hyperechoing of the fatty pancreas relative to the renal echo as its diagnostic approach. However, it lacks precision as pancreatic fibrosis produces similar hyperecho, making differentiation difficult. This is in addition to the fact that the kidney and pancreas are not visible under the same window, thereby requiring separate ultrasound of the kidney-liver and pancreas-liver for diagnosis, which makes the acceptability of the diagnosis operator-dependent [6]. The endoscopic ultrasound (EUS), though reliable and accurate with higher resolution, better imaging, and visualization, is unfit for widespread use because it involves invasive procedures and the associated risk and cost. Studies have reported several benefits of CT and MRI for their non-invasiveness, high accuracy, and sensitivity, though radiation exposure is a concern [7].

Obesity has emerged as the paramount worldwide health issue, with its prevalence rising at an unparalleled pace. It is linked to many other health problems that affect people all over the world, including diabetes, metabolic syndrome (MetS), cardiovascular and cerebrovascular diseases, mental illness, and cancer [1,8]. One out of every eight people in the globe was obese in 2022, while 2.5 billion people (18 years and older) were overweight [9]. As obesity, MetS, and type 2 diabetes become more common around the world, NAFPD is becoming a bigger clinical issue [10].

Pancreatic ductal adenocarcinoma (PDAC), the predominant and most fatal kind of pancreatic cancer, is frequently identified at an advanced stage, exacerbating its poor prognosis [11,12]. Recent studies indicate that pancreatic steatosis may function as a pre-neoplastic niche that promotes an environment favorable for developing PDAC. Lipotoxicity, chronic low-grade inflammation, and pancreatic fibrosis are suggested as mechanistic mediators in the progression from benign steatosis to cancer [13].

Wide-ranging research has examined hepatic fatty infiltration, a factor in the 30 % prevalence of non-alcoholic fatty liver disease (NAFLD). Deterioration in NAFLD may result in non-alcoholic steatohepatitis (NASH), cirrhosis, hepatic carcinoma, and even NAFPD [[14], [15], [16], [17], [18]]. Nonetheless, the epidemiology of NAFPD remains little known, presumably attributable to the paucity of recognized diagnostic criteria and insufficient knowledge among healthcare professionals. Moreover, scanty studies have synthesized scientific evidence on the epidemiological risk, metabolic–inflammatory pathways, and immune-microenvironmental alterations in NAFPD.

This review seeks to integrate contemporary clinical and molecular information connecting NAFPD to PDAC, emphasizing metabolic and inflammatory pathways that could function as early, amendable targets for diagnostic efforts and preventive and therapeutic interventions. Specifically, the study *meta*-synthesizes scientific evidence from published English-language literature between 2014 and April 2025 involving clinical cohorts, *in-vivo* and molecular studies that investigated the relationship between pancreatic steatosis and PDAC. The stratified data focus on understanding (i) epidemiological risk, (ii) metabolic–inflammatory pathways, and (iii) immune-microenvironmental alterations.

## Methodology

This evidence synthesis review was executed following a modified PRISMA-ScR guideline (Preferred Reporting Items for Systematic Reviews and Meta-Analyses Extension for Scoping Reviews) [[19], [20], [21]]. The following search terms were used to find articles in PubMed, Scopus, and Web of Science: (“non-alcoholic fatty pancreas disease” OR “pancreatic steatosis”) AND (“pancreatic cancer” OR “pancreatic ductal adenocarcinoma”) AND (“inflammation” OR “lipotoxicity” OR “metabolic syndrome”). The inclusion criteria were peer-reviewed original research published between 2014 and April 2025. The study concentrated mainly on articles reporting molecular pathways (metabolic–inflammatory, immune-microenvironmental alterations), histological, or epidemiological association evidence between NAFPD and PDAC. Articles not written in English, case reports, and/or studies that did not have original data were excluded. The study protocol was registered PROSPERO and available from https://www.crd.york.ac.uk/PROSPERO/view/CRD420251168072.

### Assessment of data quality

The data quality for this *meta*-synthesis of scientific evidence was assessed using the Newcastle-Ottawa Scale (NOS) approved by the Agency for Healthcare Research and Quality (AHRQ) (https://www.ohri.ca/programs/clinical epidemiology/oxford.asp). The quality of the studies was graded into three categories—(i) epidemiological (population (size/design), diagnostic modality for pancreatic fat) (ii) metabolic–inflammatory pathways, (event/pathway, key molecules regulated, mechanism link from steatosis to tumorigenesis), and (iii) immune-microenvironmental alterations (findings relevant to PDAC) —using a star system [21,22].

## Results synthesis and discussion

**Fig. 1:**
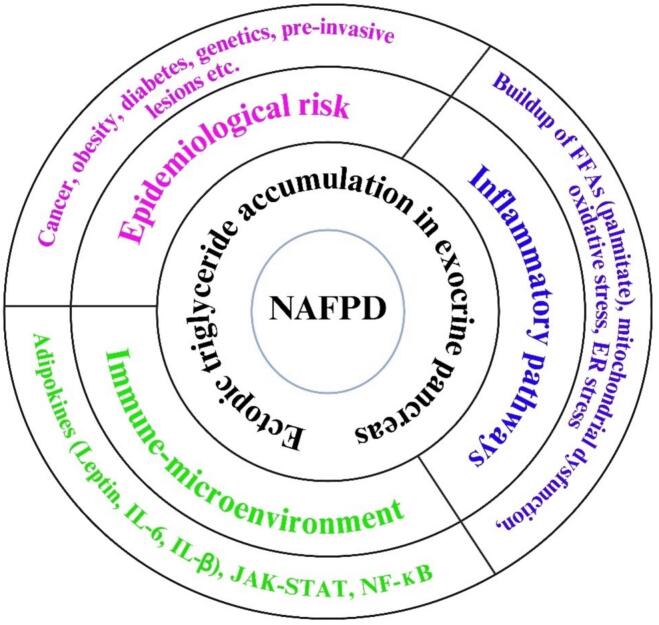
Overview of the *meta*-synthesis evidence of NAFPD as a Pre-Neoplastic Niche.

A total of 93 articles were identified from the three databases: PubMed (20 articles; 21.51 %), Scopus (28 articles; 30.11 %), and Web of Science (45 articles; 48.39 %). During the initial screening, 11 articles (11.83 %) were eliminated for not meeting the inclusion criteria, including meeting Abstract (2), editorial material (1), conference proceeding paper (2), book chapter (1), and articles written non-English languages like Czech (2), Chinese (1), Hungarian (1), and Spanish (1) (Supplementary file 1). Normalizing the data in ScientoPy and fBasics R-packages [19,21], 16 (17.20 %) duplicates were identified, removed, and saved in CSV or Excel files. After checking for relevant studies reporting epidemiological risk, metabolic–inflammatory pathways, and immune-microenvironmental changes from the title, abstract, and full text, 29 (31.18 %) were disqualified, and 37 (39.78 %) articles were chosen for the evidence synthesis detailed in S Fig. 1 of the PRISMA flowchart.

### Quality assessment

The quality evaluation of risk of bias assessment ratings of the included studies are indicated in supplementary Table S1, displaying specific information on the evaluation questions listed per domain for each article. The quality ratings for the included studies range from 4 to 6. Of the possible 6 points, three studies received 6 points, four received 5 points, and fourteen received 4 points.

**Fig. 2:**
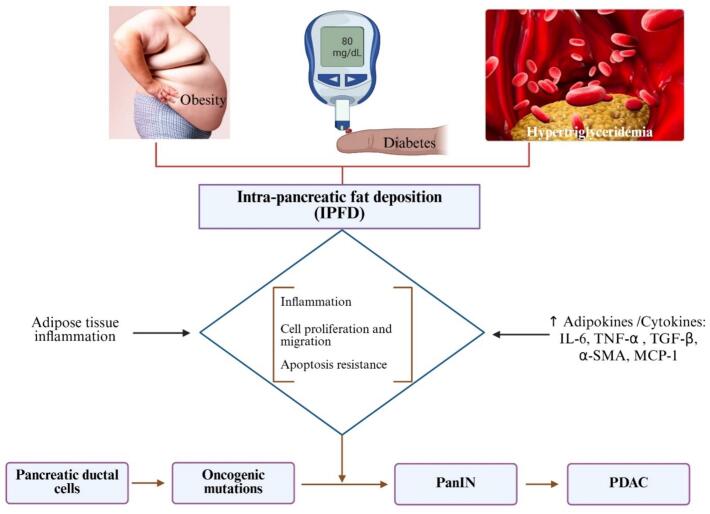
Proposed processes connecting intra-pancreatic fat deposition (IPFD) to pancreatic cancer. IL-6, interleukin-6; MCP-1, monocyte chemoattractant protein-1; PanIN, pancreatic intraepithelial neoplasia; PDAC, pancreatic ductal adenocarcinoma; α-SMA, α-smooth muscle actin; TGF-β, transforming growth factor-β; TNF-α, tumor necrosis factor-α.

ER, endoplasmic reticulum; FFA, free fatty acids; IL, interleukin; JAK-STAT, Janus Kinases – Signal Transducers and Activators of Transcription; NF-κB, Nuclear Factor kappa-light-chain-enhancer of activated B cells.

Based on the findings from the *meta*-synthesis, the key epidemiological links between NAFPD and PDAC were cancer, obesity, diabetes, genetic alterations, and pre-invasive lesions (Fig. 1). The molecular pathways include adipocyte–acinar crosstalk, fibrosis, IL-6/JAK-STAT5 inflammatory axis, lipid-droplet biogenesis, fatty acid (FA) β-oxidation, lipotoxic DNA-damage signaling, NOD-, LRR- and pyrin domain-containing protein 3 (NLRP3) inflammasome; adipokine shift, adipokine–cytokine networks, EMT, extracellular-matrix remodeling, and glucose/FA metabolic convergence (Fig. 1, Table 1, Table 2). The tumor microenvironment comprising endoplasmic reticulum (ER) stress, adipokines, and cytokines (leptin, IL-6, IL-1β) activates JAK-STAT and NF-κB pathways (Fig. 1 and Table 2). This study proposed that the processes of intra-pancreatic fat deposition (IPFD) may elevate the risk of pancreatic cancer development and progression by facilitating the release of adipokines that incite inflammation, inhibit apoptosis, and promote cell proliferation and migration (Fig. 2).

**Table 1:** Clinical evidence linking NAFPD/pancreatic steatosis to PDAC risk.

References	Population (Size/Design)	Diagnostic Modality for Pancreatic Fat	Findings Relevant to PDAC
[10]	162 consecutive EUS cases (single-centre, Jakarta)	Endoscopic ultrasound (qualitative echogenicity)	The total prevalence of fatty pancreas was 32.7 %, however, it was much greater in patients with PDAC. Multivariable analysis showed NAFPD as the sole independent risk factor for PDAC (OR = 18).
[23]	183 distal-pancreatectomy patients (75 PDAC vs 108 other)	Unenhanced CT “pancreatic index” (density ratio pancreas/spleen)	CT-defined fatty pancreas (PI ≤ 0.70) autonomously predicted PDAC (OR 2.31; P = 0.023).
[24]	55 surgical patients (24 PDAC, 31 controls)	3 T MRI-PDFF vs CT	The MRI-measured fat fraction was much greater in PDAC and was substantially linked to histology (r = 0.80). MRI-PDFF was better than CT at finding steatosis linked to cancer.
[25]	Retrospective case-control: 32 future-PDAC cases vs 117 matched controls	Non-contrast CT (pancreas-to-spleen HU ratio < 0.70)	Steatosis was observed ≤ 3 years before to diagnosis in 72 % of cases compared to 45 % of controls (adjusted OR 2.7). Indicates that pancreatic fat serves as an early imaging biomarker for PDAC.
[26]	UK-Biobank cohort (n = 29 463; median follow-up 4.5 y)	Dixon MRI quantification of IPFD	Severe IPFD (> 10 %) increased the probability of incident PDAC threefold (HR = 3.0), with Mendelian randomization corroborating causality.
[27]	17 clinical studies, 5 456 PDAC patients	Mixed CT/MRI/EUS	The combined prevalence of pancreatic steatosis in PDAC is 53.6 %, and the combined odds ratio for steatosis compared to controls is 3.23 (95 % CI 1.86–5.60).
[28]	187 resected PDAC patients	Contrast CT density + histopathologic fat quantification	Fatty pancreas predicted increased surgical morbidity.
[29]	234 surgical & screening subjects (68 PDAC, 166 controls)	Non-contrast CT attenuation index < 0.8	Fat infiltration independently elevated PDAC risk.
[30]	Risk subjects in a surveillance programme	Volumetric CT fat segmentation	The pancreatic fat accumulation in the upper tertile increased the incidence of PDAC.

**Table 2:** Molecular events that mechanistically bridge non-alcoholic fatty-pancreas disease (NAFPD)/pancreatic steatosis to pancreatic-ductal-adenocarcinoma (PDAC).

Event/pathway	Key molecules (↑ = up-regulated; ↓ = down-regulated)	Mechanistic link from steatosis → tumorigenesis	References
Lipotoxic ER & oxidative stress	↑ Palmitate, oleate, ROS, PERK–eIF2α–CHOP, γ-H2AX	FFA overload causes ER stress–driven apoptosis/compensatory proliferation and DNA damage that initiates PanIN lesions	[26,31]
Adipokine dysregulation	↑ Leptin, resistin, IGF-1; ↓ Adiponectin	Leptin/JAK2–STAT3 and IGF-1/PI3K-AKT enhance ADM and KRAS fitness	[[30], [32], [33]]
Inflammasome cytokine loop	↑ NLRP3, caspase-1, IL-1β, IL-6, TNF-α; NF-κB p65	Lipotoxic acini trigger NLRP3–IL-1β → paracrine IL-6; activates JAK-STAT3/5 in ducts, sustaining chronic inflammation	[[10], [34]]
Metabolic re-programming	↑ SREBP1c, FASN, CPT1A, PGC-1α	KRAS < sup > G12D</sup > suppresses HSL, drives lipid droplets accumulation; fatty acid β-oxidation supplies NADPH & ATP for tumor growth	[35]
Fibrogenic stellate-cell activation	↑ TGF-β1, SMAD2/3, α-SMA, collagen I/III	Steatosis-induced ROS and TGF-β activate PSCs, stiffening the ECM and facilitating ADM/EMT.	[[31], [36]]
ECM remodeling and stiffness	↑ LOX, MMP-9, TIMP-1, hyaluronan	Cross-linked collagen increases interstitial pressure and augments integrin-FAK signaling and invasiveness.	[[37], [38]]
Oncogenic driver synergy	Mutant KRAS, p53 < sup > mut</sup>, CDKN2A < sup > loss</sup>; ↑ STAT5	Steatosis accelerates KRAS-mediated PanIN progression; STAT5 ablation delays PDAC in fatty-pancreas mice	[[30], [32]]
Epigenetic/miRNA modulation	↑ miR-21, miR-155; ↓ miR-217, HDAC7 acetyl-marks	FFAs alter miRNA profiles and histone acetylation, silencing tumor suppressors and promoting EMT.	[[39], [40]]
Immune-checkpoint re-wiring	↑ PD-L1, PD-L2 on ductal cells & PSCs; M2-TAM polarization	Lipid-laden PSCs secrete CXCL1/8 recruiting M2 macrophages; PD-L1 dampens CD8 < sup>+</sup > T-cell cytotoxicity	[[34], [41]]
Angiogenesis/hypoxia	↑ VEGF-A, HIF-1α, ANGPTL4	Hypoxic fatty stroma up-regulates VEGF; vascular leakiness aids tumor expansion and early dissemination.	[29]

ADM, acinar-to-ductal metaplasia; Akt, protein kinase B; ANGPTL4, angiopoietin-like 4; ATP, adenosine triphosphate; CHOP, CCAAT/enhancer-binding protein homologous protein; CPT1A, carnitine palmitoyltransferase 1A; CXCL, Chemokine (C-X-C motif) ligand; DNA, deoxyribonucleic acid; ECM, extracellular matrix; EMT, epithelial-mesenchymal transition; eIF2α, eukaryotic initiation factor 2α; ER, endoplasmic reticulum; FAK, focal adhesion kinase; FASN, fatty acid synthase; FFA, free fatty acid; γ-H2AX, gamma phosphorylated version of histone protein; HDAC, histone deacetylse; HIF-1α, hypoxia-inducible factor 1-alpha; HSL, hormone sensitive lipase; IGF, inculin-like growth factor; IL, interleukin; JAK-STAT, Janus Kinases – Signal Transducers and Activators of Transcription; KRAS, Kirsten rat sarcoma virus; LOX, lysyl-oxidase; MMP, matrix metalloproteinase; NADPH, nicotinamide adenine dinucleotide phosphate; NF-κB, Nuclear Factor kappa-light-chain-enhancer of activated B cells; NLRP3, pyrin domain-containing protein 3; PanIN, pancreatic intraepithelial neoplasia; PDAC, pancreatic ductal adenocarcinoma; PD-L, programmed death-ligand; PERK, protein kinase R-like endoplasmic reticulum kinase; PGC-1α, Peroxisome proliferator-activated receptor-γ coactivator 1α; PI3K, phosphoinositide 3-kinase; PSC, pancreatic stellate cell; ROS, reactive oxygen species; SMA, smooth muscle actin; SMAD, small mothers against decapentaplegic protein; SREBP1c, Sterol Regulatory Element Binding Protein-1c; TAM, tumor-associated macrophages; TGF, transforming growth factor; TIMP, tissue inhibitor of metalloproteinases; TNF, tumor necrosis factor; VEGF, vascular endothelial growth factor.

### Evidence from epidemiological and clinical data linking NAFPD to PDAC risk

The scientific evidences from several articles, based on hospital data, retrospective cohort confirmations, a multi-ethnic cohort of MRI, Mendelian randomization, and endoscopic-ultrasound biopsy, suggest epidemiological links between NAFPD and PDAC [10,[25], [26], [29], [36]]. These epidemiological links include cancer, obesity and diabetes, fat accumulation, genetic alterations, and fatty pancreas. The increasing population- and hospital-based data reveal that NAFPD is more than a mere incidental radiological finding; it is an independent, dose-responsive risk factor for PDAC. Cross-sectional imaging studies initially demonstrated that steatosis is significantly over-represented in cancer patients: moderate-to-severe fat infiltration was observed in 46 % of PDAC cases compared to 21 % of non-cancer controls in a pooled *meta*-analysis of 2,956 individuals (OR = 2.48, 95 % CI = 1.85–3.32) [27]. Extensive retrospective cohorts validate the correlation; among 5642 computed tomography (CT)-screened individuals, pancreatic fat in the highest tertile resulted in a two-fold elevation in the incidence of PDAC adjusted for body mass index, diabetes, and alcohol consumption [19,29]. Temporal evidence also fortifies the association. Hoogenboom et al. [20] demonstrated that steatosis was apparent on CT up to three years before diagnosis of PDAC, whereas the baseline pancreatic fat volume tripled the five-year cancer incidence in high-risk surveillance individuals [7]. A prospective multi-ethnic cohort utilizing MRI and Mendelian randomization indicated a 36 % increase in PDAC hazard per standard deviation rise in fat fraction, suggesting a genetically established causal association [26].

In addition to risk assessment, NAFPD forecasts clinical outcomes. In a cohort of 187 resected PDAC patients, a fatty pancreas independently increased postoperative morbidity and one-year mortality [36,27]. Endoscopic-ultrasound biopsy studies have established a connection between steatosis and pre-invasive PanIN and intraductal papillary mucinous neoplasms (IPMN) lesions [7,21,[25], [26], [29], [42]], identifying NAFPD as an early, modifiable entity within the neoplastic continuum. A higher number of cancer patients had steatosis. Moderate to severe pancreatic fat is detected in almost half of PDAC cases and one-fifth of controls [29], showing a pooled odds ratio of about 2.5 (95 % CI 1.9–3.3). The Mendelian randomization connects genetically predicted pancreatic fat to a 36 % increase in PDAC risk for every standard deviation (SD) rise in fat fraction, reducing any remaining confounding factors [27]. These epidemiological indicators validate the integration of quantitative pancreatic fat measures into PDAC risk models and highlight the necessity for longitudinal cohorts to evaluate whether fat reduction decreases cancer incidence.

The findings of the last ten years depict that clinical studies have repeatedly shown that NAFPD, which is typically seen as pancreatic steatosis on cross-sectional imaging, is a new, separate risk factor for PDAC (Table 1). There is uniformity across modalities and demographics among the studies. CT-based density indices, MRI-proton-density-fat-fraction, and qualitative EUS, all show that PDAC cohorts had more fat, and the associations endured after adjustments for obesity, diabetes, and age [25,23]. The Mayo Clinic study demonstrates that steatosis can precede clinical PDAC by as much as three years, hence reinforcing the case for causality [25]. Extensive MRI data demonstrate a relationship between dose and response, with a threshold effect demonstrating that at least 10 % intra-pancreatic fat triples risk of PDAC [26]. Molecular mechanistic plausibility shows that lipotoxicity encourages long-term inflammation, acinar-ductal metaplasia, and Kirsten rat sarcoma virus (KRAS)-mediated cancer growth. Clinical investigations adding pancreatic fat measurement to high-risk surveillance algorithms should help better classify PDAC, especially in patients who are obese or have metabolic syndrome. MRI-proton density fat fraction (PDFF) provides a radiation-free quantitative biomarker, although non-contrast CT metrics can be opportunistically obtained from standard abdomen scans.

Furthermore, several extensive imaging and histological investigations have indicated a substantial correlation between pancreatic adiposity and PDAC risk. Imaging investigations employing MRI and CT scans have demonstrated augmented pancreatic fat accumulation in individuals at elevated risk for PDAC. The study by Papalamprakopoulou et al. [43] revealed that individuals with NAFPD often present with premalignant histopathological abnormalities, including PanIN and IPMN. Likewise, a greater incidence of PDAC in people exhibiting steatotic pancreas was identified using CT scans [30]. Moreover, pancreatic steatosis has been associated with unfavorable postoperative outcomes in PDAC patients following surgical resection. Zhou et al. [30] additionally presented histological evidence indicating that patients undergoing PDAC surgery with concurrent NAFPD have inferior surgical results, characterized by heightened inflammatory complications and reduced survival rates. The combination of these shreds of scientific evidence indicates that fatty infiltration of the pancreas not only signifies metabolic stress but also exacerbates PDAC prognosis.

CI, confidence interval; CT, computed tomography; EUS, endoscopic ultrasound; HU, Hounsfield units; IPFD, intra-pancreatic fat deposition; MRI, magnetic resonance imaging; NAFPD, non-alcoholic fatty pancreatic disease; PDAC, pancreatic ductal adenocarcinoma; PDFF, proton density fat fraction; PI, pancreatic index.

### Molecular pathways: From steatosis to tumorigenesis

Pancreatic steatosis triggers a series of metabolic stress events at the molecular level that pave the way for malignant transformation. Lipotoxicity caused by the excessive buildup of free fatty acids (FFAs), particularly palmitate, results in mitochondrial dysfunction, oxidative stress, and endoplasmic reticulum (ER) stress [44]. These stressors increase the activity of transcription factors, including nuclear factor-kappa B (NF-κB) and STAT3, which then cause the release of pro-inflammatory cytokines like IL-6, TNF-α, and MCP-1 [45]. Chronic inflammation also activates stellate cells and fibrogenesis, which are known to lead to desmoplasia, a sign of PDAC. Recent transcriptome investigations have revealed the overexpression of genes such as KRAS, tumor protein P53 (TP53), and cyclin-dependent kinase inhibitor 2A (CDKN2A) in steatotic pancreatic settings, suggesting a transition towards an oncogenic landscape even before the manifestation of visible tumors [46].

**Fig. 3:**
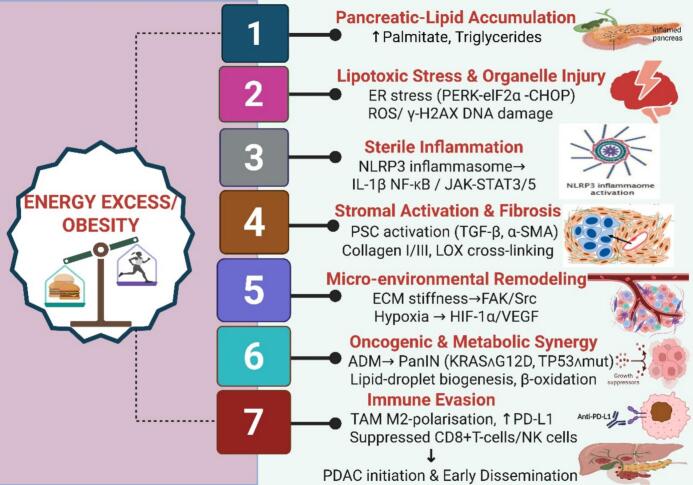
A hypothesis molecular mechanistic cascade of events from NAFPD to PDAC.

The lipid accumulation into the pancreas, involving chronic caloric surplus and insulin resistance, facilitates the accumulation of palmitate-enriched triglycerides in acinar and inter-lobular regions, characterizing NAFPD [25] as hypothesized in Fig. 3, and followed by lipotoxic endoplasmic reticulum and oxidative stress. It involves saturated fatty acids that stimulate the protein kinase R-like endoplasmic reticulum kinase (PERK)– eukaryotic initiation factor 2α (eIF2α)– CCAAT/enhancer-binding protein homologous protein (CHOP) pathway and produce reactive oxygen species (ROS), resulting in gamma phosphorylated version of histone protein H2A (γ-H2AX) DNA breaks and initiating the process of acinar-to-ductal metaplasia [31]. The inflammation that is sterile, which is characterized by the damaged acini, sends out danger signals that get NLRP3 ready; caspase-1 cuts pro-IL-1β, and lipids cause NF-κB and IL-6 to be released, which creates a self-amplifying cytokine–adipokine loop [47]. Activation of fibrogenic stellate cells, i.e., transforming growth factor-beta (TGF-β) and oxidative stress, transforms quiescent pancreatic stellate cells (PSCs) into α-smooth muscle actin (α-SMA)^+^ myofibroblasts that secrete collagen I/III; lysyl-oxidase (LOX) cross-linking enhances the rigidity of the matrix [48,38], thereby inducing changes in biomechanics and metabolism. Integrin-focal adhesion kinase (FAK)/Src signaling is turned on when the extracellular matrix (ECM) stiffens. Hypoxia in the bulky fatty stroma, on the other hand, stabilizes hypoxia-inducible factor 1-alpha (HIF-1α) and vascular endothelial growth factor (VEGF), speeding up angiogenesis and invasion [29]. That could lead to oncogenic cooperation with KRAS. In Kras^G12D mice on a high-fat diet, intrapancreatic fat promotes PanIN and PDAC through STAT5-dependent transcription and inhibition of hormone-sensitive lipase, therefore confining cells to a lipid-rich, β-oxidative phenotype [35,32]. Finally, there is the evasion of the immune system, where the lipid-rich PSCs release CXCL1/8, which draws in M2-polarized macrophages. On the other hand, ductal cells increase programmed death-ligand 1 (PD-L1), which slows down the activity of cytotoxic T-cells [[34], [41], [49]] (Fig. 3).

Steatosis collectively provides the metabolic fuel, inflammatory signals, and stromal environment that reduce the threshold for KRAS-driven transformation and facilitate the early dissemination of malignant cells. Interventions that diminish pancreatic fat (GLP-1 agonists), inhibit IL-6/STAT3/5, or regulate PSC activation provide viable ways to disrupt this cascade. A summary of findings from the studies that reported the molecular pathways from steatosis to tumour formation is presented in Table 2.

ADM, acinar-to-ductal metaplasia; CHOP, CCAAT/enhancer-binding protein homologous protein; ECM, extracellular matrix; eIF2α, eukaryotic initiation factor 2α; ER, endoplasmic reticulum; FAK, focal adhesion kinase; γ-H2AX, gamma phosphorylated version of histone protein; HIF-1α, hypoxia-inducible factor 1-alpha; IL-1β, interleukin 1 beta; JAK-STAT, Janus Kinases – Signal Transducers and Activators of Transcription; LOX, lysyl-oxidase; NF-κB, Nuclear Factor kappa-light-chain-enhancer of activated B cells; NLRP3, pyrin domain-containing protein 3; PanIN, pancreatic intraepithelial neoplasia; PDAC, pancreatic ductal adenocarcinoma; PD-L1, programmed death-ligand 1; PERK, protein kinase R-like endoplasmic reticulum kinase; PSC, pancreatic stellate cell; ROS, reactive oxygen species; SMA, α-smooth muscle actin; TAM, tumor-associated macrophages; TGF-β, transforming growth factor-beta; VEGF, vascular endothelial growth factor.

### NAFPD-induced pancreatic exocrine atrophy

It is well-established that the fat accumulation in the pancreas in patients with NAFPD leads to death of pancreatic acinar cells, which are consequently replaced by adipocytes – a phenomenon called fatty replacement [50]. This replacement causes pancreatic atrophy (also called pancreatic lipomatosis) and consequently leads to exocrine pancreas insufficiency (EPI), where there is deficient secretion of the pancreatic digestive enzymes, thereby severing the digestive and metabolic processes dependent on the pancreatic enzymes. Some consequences of the ensuing maldigestion and malabsorption of nutrients may include diarrhoea, bloating, fatty stools, and weight loss. Risk factors for atrophy include viral infection, iron deposition, drug-induced injury, pancreatic duct obstruction, and obesity and metabolic syndrome that elicit the NADPD [45]. This phenotype is different from NAFPD without pancreatic exocrine atrophy, where fat accumulation does not cause significant replacement of the exocrine pancreatic tissues, and thus, the structure and function of the exocrine pancreas remain intact. In other words, this type's symptoms of maldigestion and malabsorption are absent [51]. Two metabolic bariatric surgery methods, including sleeve gastrectomy and Roux-en-Y-gastric bypass, are the most common and effective interventions for morbid obesity, resulting in significant weight loss and improvement in obesity-related metabolic disorders [52]. Endoscopic bariatric therapy is also offered to obese patients who are not fit for metabolic bariatric surgery. Studies have reported changes in intra-pancreatic fat deposition following bariatric surgeries [53,54]. This supports the contention that effective management of obesity and other metabolic syndromes can be a strategy to prevent or control NAFPD and its consequent PDAC.

### Co-existence of NAFPD with diabetes

The co-existence of NAFPD with insulin resistance and diabetes has been well-reported in several articles [2]. The inflammatory response from the adipokines produced from excessively accumulated fat in the pancreas of patients with NADPD has toxic effects, not only on the acinar cells, but also on the islet cells that produce glucose-regulating hormones, thereby causing insulin resistance and diabetes mellitus [55,56]. Some studies have reported accumulation of fat in the pancreas of diabetic patients [57,58]. A cross-sectional study in South Korea showed an increase in Homeostatic Model Assessment for Insulin Resistance (HOMA-IR) and other symptoms of metabolic syndrome among patients with NAFPD [6]. Another cross-sectional study in Germany among healthy Caucasians with increased risk of type 2 diabetes showed that pancreatic fat content negatively correlates with insulin secretion [59]. This shows that, in addition to obesity, NAFPD may co-exist with diabetes mellitus. The mechanism for NAFPD-induced β-cell dysfunction is glucolipotoxicity. For instance, hyperglycemia inhibits carnitine palmitoyl transferase 1 in the β-cell by increasing malonyl coenzyme A, decreasing mitochondrial β-oxidation, and consequently augmenting intracellular triglyceride accumulation. This is strengthened as the inhibitory effect of insulin on peripheral lipolysis is decreased by insulin resistance. Together, these lead to chronic exposure of β-cells to FFAs, leading to decreased insulin gene expression and blunted insulin-stimulated glucose secretion [2].

Glucagon-like peptide (GLP-1) is a polypeptide hormone secreted by the L-cells in the terminal ileum and colon, and it promotes insulin secretion via the incretin effect [60] and enhances peripheral tissue sensitivity to insulin [61]. The incretin effect is reduced in diabetic and obese patients, both of whom tend to develop NAFPD [62]. Similarly, a study reported a decrease in serum GLP-1 levels in NAFPD patients compared to normal subjects, demonstrating that altered GLP-1 regulation is an independent risk factor for pancreatic steatosis [3]. Thus, GLP-1 is a promising therapeutic target for controlling the metabolic risk factors associated with the development of NAFPD. This speculation is supported by a recent finding that semaglutide (a GLP-1 receptor agonist) promotes weight loss, reduces adipocyte size and macrophage infiltration, stimulates browning of adipocytes, improves mitochondrial biogenesis, and reduces ER stress [63].

### Limitations and recommendations for further studies

One of the major limitations of this study is that most of the epidemiological and clinical data in the studies reviewed are associative rather than causative, thereby making it very difficult to conclude that NAFPD causes PDAC. While our *meta*-synthesis shows differences and gaps in knowledge, the reported odds ratios range from 1.9 to 3.1 due to varying fat thresholds, ethnic diversity, and confounder control. Standardizing imaging cut-offs and validating them in longitudinal, population-based screenings is essential. Future interventional trials, such as those targeting GLP-1 or SGLT2 pathways for fat reduction, may elucidate whether modifications in steatosis reduce the prevalence of PDAC. The scientific evidence supports the integration of quantitative pancreatic-fat measurements into risk-stratified PDAC screening algorithms, particularly for patients who already fulfil high-risk criteria (familial history, IPMN, new-onset diabetes). Multicenter registries that standardize MRI/CT methods and investigate the metabolomic markers of lipotoxic damage will be essential in subsequent stages. Most studies are retrospective and employ inconsistent definitions of fat. Thus, prospective, multi-ethnic cohorts with long-term imaging and mechanistic trials that evaluate fat-modifying treatments (such as bariatric surgery [weight loss] or GLP-1 agonists) on the number of cases of PDAC are needed. Furthermore, future research should concentrate on longitudinal cohort studies validating NAFPD as a PDAC risk biomarker, clarifying cell-specific transcriptional alterations within the fatty pancreas, and evaluating anti-steatotic and anti-inflammatory medicines in NAFPD-PDAC models.

### Conclusion

The scientific findings show that NAFPD should not be considered a benign bystander. NAFPD exhibits features of a pre-neoplastic milieu, encompassing metabolic stress, chronic inflammation, stromal remodeling, and immune evasion. Its molecular functions in creating a pro-tumorigenic metabolic and inflammatory environment indicate its significance as a pre-neoplastic state. Because it can be changed by losing weight, controlling blood sugar, and taking anti-inflammatory drugs, NAFPD is an important point of intervention for preventing PDAC. Interventions that reduce pancreatic fat or block IL-6/STAT and stellate-cell activation should be clinically evaluated as strategies for PDAC prevention.

Clinical trial number

Not applicable

Ethical approval

Not applicable

## Consent to participate

Not applicable

## Consent to publish

All the authors have read and agreed to the final copy of the manuscript.

Funding

This research was supported by the National Research Foundation (NRF) of South Africa under the SARChI Chair program (Grant No. PPNT230823145247), awarded to Prof. Yahya Choonara.

## CRediT authorship contribution statement

**Hope Onohuean:** Writing – review & editing, Writing – original draft, Visualization, Validation, Software, Methodology, Investigation, Formal analysis, Data curation, Conceptualization. **Ngozi F. Nnolum-Orji:** Writing – original draft, Validation, Investigation, Formal analysis, Data curation. **Sarad Pawar Naik Bukke:** Writing – review & editing, Validation, Resources, Project administration, Methodology, Investigation, Formal analysis, Data curation. **Kasim Sakran Abass:** Writing – review & editing, Validation, Software, Resources, Methodology, Investigation, Formal analysis. **Abdullateef Isiaka Alagbonsi:** Writing – review & editing, Visualization, Resources, Methodology, Investigation, Formal analysis, Data curation. **Yahya E. Choonara:** Writing – review & editing, Validation, Resources, Project administration, Methodology, Investigation, Formal analysis, Data curation.

## Declaration of competing interest

The authors declare that they have no known competing financial interests or personal relationships that could have appeared to influence the work reported in this paper.

## References

[1] Zhang C.L., Wang J.J., Li J.N., Yang Y. (2021). Nonalcoholic fatty pancreas disease: an emerging clinical challenge. World J Clin Cases.

[2] Yu T.Y., Wang C.Y. (2017). Impact of non-alcoholic fatty pancreas disease on glucose metabolism. J Diabetes Investig.

[3] Weng S, Zhou J, Chen X, Sun Y, Mao Z, Chai K. Prevalence and factors associated with nonalcoholic fatty pancreas disease and its severity in China. Med (United States) 2018. https://doi.org/10.1097/MD.0000000000011293. 10.1097/MD.0000000000011293 PMC6039627 29953011

[4] Singh R.G., Yoon H.D., Wu L.M., Lu J., Plank L.D., Petrov M.S. (2017). Ectopic fat accumulation in the pancreas and its clinical relevance: a systematic review, meta-analysis, and meta-regression. Metabolism.

[5] Lee Y., Lingvay I., Szczepaniak L.S., Ravazzola M., Orci L., Unger R.H. (2010). Pancreatic steatosis: Harbinger of type 2 diabetes in obese rodents. Int J Obes.

[6] Lee J.S., Kim S.H., Jun D.W., Han J.H., Jang E.C., Park J.Y. (2009). Clinical implications of fatty pancreas: correlations between fatty pancreas and metabolic syndrome. World J Gastroenterol.

[7] Pang C., Dong P., Yang J., Fan Z., Cheng Z., Zhan H. (2024). Non-alcoholic fatty pancreas disease: an updated review. J Pancreatol.

[8] Passarello K., Kurian S., Villanueva V. (2019). Endometrial cancer: an overview of pathophysiology, management, and care. Semin Oncol Nurs.

[9] WHO. Obesity and overweight 2025. https://www.who.int/news-room/fact-sheets/detail/obesity-and-overweight (accessed June 17, 2025).

[10] Lesmana C.R.A., Gani R.A., Lesmana L.A. (2018). Non-alcoholic fatty pancreas disease as a risk factor for pancreatic cancer based on endoscopic ultrasound examination among pancreatic cancer patients: a single-center experience. JGH Open.

[11] Onohuean H., Adisa R.A., Alagbonsi A.I. (2021). Anti-apoptotic effect of Buchholzia coriacea Engl . stem back extracts on AsPC-1 and mechanisms of actio. BMC Complement Med Ther.

[12] Wood L.D., Canto M.I., Jaffee E.M., Simeone D.M. (2022). Pancreatic cancer: pathogenesis, screening, diagnosis, and treatment. Gastroenterology.

[13] Tarantino G., Savastano S., Colao A. (2010). Hepatic steatosis, low-grade chronic inflammation and hormone/growth factor/adipokine imbalance. World J Gastroenterol.

[14] Hales CM, Carroll MD, Fryar CD, Ogden CL. Prevalence of Obesity Among Adults and Youth: United States, 2015-2016. NCHS Data Brief 2017. 29155689

[15] Wang Y., Beydoun M.A., Min J., Xue H., Kaminsky L.A., Cheskin L.J. (2021). Has the prevalence of overweight, obesity and central obesity levelled off in the United States? Trends, patterns, disparities, and future projections for the obesity epidemic. Int J Epidemiol.

[16] Buckholz A., Mathews S.N., Ghosh G., Rosenblatt R.E., Jesudian A., Fortune B.E. (2019). Sa1621 – health and demographic disparities in Nafld and advanced fibrosis among latinos. Gastroenterology.

[17] Le M.H., Devaki P., Ha N.B., Jun D.W., Te H.S., Cheung R.C. (2017). Prevalence of non-alcoholic fatty liver disease and risk factors for advanced fibrosis and mortality in the United States. PLoS One.

[18] Rafique M., Kristjansson D. (2021). Determinants of hepatocellular carcinoma in the United States: differences in risk factor and genetic susceptibility by race/ethnicity. Georg Sci Res J.

[19] Onohuean H., Oosthuizen F. (2025). The burden of unlawful use of opioid and associated epidemiological characteristics in Africa: a scoping review. PLoS One.

[20] Tricco A.C., Lillie E., Zarin W., O’Brien K.K., Colquhoun H., Levac D. (2018). PRISMA extension for scoping reviews (PRISMA-ScR): checklist and explanation. Ann Intern Med.

[21] Onohuean H., Oosthuizen F. (2023). Multinational appraisal of the epidemiological distribution of opioid fatalities: a systematic review and meta-analysis. Front Psych.

[22] Onohuean H., Olot H., Onohuean F.E., Bukke S.P.N., Akinsuyi O.S., Kade A. (2025). A scoping review of the prevalence of antimicrobial-resistant pathogens and signatures in ready-to-eat street foods in Africa: implications for public health. Front Microbiol.

[23] Fukuda Y., Yamada D., Eguchi H., Hata T., Iwagami Y., Noda T. (2017). CT density in the pancreas is a promising imaging predictor for pancreatic ductal adenocarcinoma. Ann Surg Oncol.

[24] Fukui H., Hori M., Fukuda Y., Onishi H., Nakamoto A., Ota T. (2019). Evaluation of fatty pancreas by proton density fat fraction using 3-T magnetic resonance imaging and its association with pancreatic cancer. Eur J Radiol.

[25] Hoogenboom S.A., Bolan C.W., Chuprin A., Raimondo M.T., van Hooft J.E., Wallace M.B. (2021). Pancreatic steatosis on computed tomography is an early imaging feature of pre-diagnostic pancreatic cancer: a preliminary study in overweight patients. Pancreatology.

[26] Yamazaki H., Streicher S.A., Wu L., Fukuhara S., Wagner R., Heni M. (2024). Evidence for a causal link between intra-pancreatic fat deposition and pancreatic cancer: a prospective cohort and Mendelian randomization study. Cell Reports Med.

[27] Vlăduț C., Steiner C., Löhr M., Gökçe D.T., Maisonneuve P., Hank T. (2025). High prevalence of pancreatic steatosis in pancreatic cancer patients: a meta-analysis and systematic review. Pancreatology.

[28] Zhou L., Xiao W.M., Li C.P., Gao Y.W., Gong W.J., Lu G.T. (2021). Impact of fatty pancreas on postoperative pancreatic fistulae: a meta-analysis. Front Oncol.

[29] Desai V., Patel K., Sheth R., Barlass U., Chan Y.M., Sclamberg J. (2020). Pancreatic fat infiltration is associated with a higher risk of pancreatic ductal adenocarcinoma. Visc Med.

[30] Chan C.H., Chang C.C., Peng Y.C. (2024). The clinical significance of pancreatic steatosis in pancreatic cancer: a hospital-based study. Diagnostics.

[31] Frendi S., Martineau C., Cazier H., Nicolle R., Chassac A., Albuquerque M. (2024). Role of the fatty pancreatic infiltration in pancreatic oncogenesis. Sci Rep.

[32] Philip B, Roland CL, Daniluk J, Liu Y, Chatterjee D, Gomez SB, et al. A High-Fat Diet Activates Oncogenic Kras and COX2 to Induce Development of Pancreatic Ductal Adenocarcinoma in Mice. Gastroenterology 2013;145:10.1053/j.gastro.2013.08.018. Doi: 10.1053/J.GASTRO.2013.08.018. 10.1053/j.gastro.2013.08.018 PMC3873752 23958541

[33] Fukuda A., Wang S.C., Morris J.P., Folias A.E., Liou A., Kim G.E. (2011). Stat3 and MMP7 contribute to pancreatic ductal adenocarcinoma initiation and progression. Cancer Cell.

[34] Wang Z., He R., Dong S., Zhou W. (2023). Pancreatic stellate cells in pancreatic cancer: as potential targets for future therapy. Front Oncol.

[35] Zhang R., Peng X., Du J.X., Boohaker R., Estevao I.L., Grajeda B.I. (2023). Oncogenic KRASG12D reprograms lipid metabolism by upregulating SLC25A1 to drive pancreatic tumorigenesis. Cancer Res.

[36] Fukuda Y., Koga C., Minami S., Ishikawa S., Gakuhara A., Fukuda S. (2025). Pancreatic fat accumulation impacts postoperative survival in patients with pancreatic ductal adenocarcinoma. World J Surg.

[37] Liu Y.H., Hu C.M., Hsu Y.S., Lee W.H. (2022.1–10.). Interplays of glucose metabolism and KRAS mutation in pancreatic ductal adenocarcinoma. Cell Death Dis.

[38] Tang H.R., Leung L., Saturno G., Viros A., Smith D., Leva D.I. (2017). Lysyl oxidase drives tumour progression by trapping EGF receptors at the cell surface. Nat Commun.

[39] Chen Y.J., Wang W.H., Wu W.Y., Hsu C.C., Wei L.R., Wang S.F. (2017). Novel histone deacetylase inhibitor AR-42 exhibits antitumor activity in pancreatic cancer cells by affecting multiple biochemical pathways. PLoS One.

[40] Toste P.A., Li L., Kadera B.E., Nguyen A.H., Tran L.M., Wu N. (2015). p85α is a microRNA target and affects chemosensitivity in pancreatic cancer. J Surg Res.

[41] Van Audenaerde J.R.M., Dev Waele J., Marcq E., Van Loenhout J., Lion E., Van den Bergh J.M.J. (2017). Interleukin-15 stimulates natural killer cell-mediated killing of both human pancreatic cancer and stellate cells. Oncotarget.

[42] Norose T., Ohike N., Suzuki R., Shibata H., Imai H., Isobe T. (2016). A Case of high-grade panin (Carcinoma In Situ) concomitant with intraductal papillary mucinous neoplasm of the pancreas: can a focal fatty change hint at peripheral panin?. J Pancreas.

[43] Papalamprakopoulou Z., Dey P., Frascati R., Fountzilas C. (2025). Pancreatic steatosis as a risk factor for pancreatic ductal adenocarcinoma: pathogenesis and clinical implications. Clin Transl Gastroenterol.

[44] Wang C.Y., Ou H.Y., Chen M.F., Chang T.C., Chang C.J. (2014). Enigmatic ectopic fat: prevalence of nonalcoholic fatty pancreas disease and its associated factors in a Chinese population. J Am Heart Assoc.

[45] Pagkali A., Makris A., Brofidi K., Agouridis A.P., Filippatos T.D. (2024). Pathophysiological mechanisms and clinical associations of non-alcoholic fatty pancreas disease. Diabetes, Metab Syndr Obes.

[46] Xiang H., Yang R., Tu J., Xi Y., Yang S., Lv L. (2023). Metabolic reprogramming of immune cells in pancreatic cancer progression. Biomed Pharmacother.

[47] Sokolova M., Sahraoui A., Høyem M., Øgaard J., Lien E., Aukrust P. (2018). Nlrp3 inflammasome mediates oxidative stress-induced pancreatic islet dysfunction. Am J Physiol - Endocrinol Metab.

[48] Tang H., Leung L., Saturno G., Viros A., Smith D., Di L.G. (2019). Author Correction: Lysyl oxidase drives tumour progression by trapping EGF receptors at the cell surface. Nat Commun.

[49] Francescone R., Vendramini-Costa D.B., Franco-Barraza J., Wagner J., Muir A., Gabitova L. (2019). Abstract 2038: NG1/NGL1 engagement supports PDAC development via CAF to PDAC nutrition and CAF-regulated immunosuppression. Cancer Res.

[50] Pinte L., Balaban D.V., Băicuş C., Jinga M. (2019). Non-alcoholic fatty pancreas disease - practices for clinicians. Rom J Intern Med.

[51] Habas E., Farfar K., Habas E., Rayani A., Elzouki A.N. (2024). Extended review and updates of nonalcoholic fatty pancreas disease. Saudi J Med Med Sci.

[52] Angrisani L., Santonicola A., Iovino P., Vitiello A., Zundel N., Buchwald H. (2017). Bariatric Surgery and Endoluminal Procedures: IFSO Worldwide Survey 2014. Obes Surg.

[53] Gaborit B., Abdesselam I., Kober F., Jacquier A., Ronsin O., Emungania O. (2015). Ectopic fat storage in the pancreas using 1 H-MRS: Importance of diabetic status and modulation with bariatric surgery-induced weight loss. Int J Obes.

[54] Wang Y., Liu Y., Petrov M.S. (2025). The effects of metabolic bariatric surgery on intra-pancreatic fat deposition and total pancreas volume: a systematic review and meta-analysis. Obes Surg.

[55] Gerst F., Wagner R., Oquendo M.B., Siegel-Axel D., Fritsche A., Heni M. (2019). What role do fat cells play in pancreatic tissue?. Mol Metab.

[56] Zhao Z.Z., Xin L.L., Xia J.H., Yang S.L., Chen Y.X., Li K. (2015). Long-term high-fat high-sucrose diet promotes enlarged islets and β-cell damage by oxidative stress in Bama Minipigs. Pancreas.

[57] Szczepaniak L.S., Victor R.G., Mathur R., Nelson M.D., Szczepaniak E.W., Tyer N. (2012). Pancreatic steatosis and its relationship to β-cell dysfunction in humans: racial and ethnic variations. Diabetes Care.

[58] Tushuizen M.E., Bunck M.C., Pouwels P.J., Bontemps S., Van Waesberghe J.H.T., Schindhelm R.K. (2007). Pancreatic fat content and beta-cell function in men with and without type 2 diabetes. Diabetes Care.

[59] Heni M., Machann J., Staiger H., Schwenzer N.F., Peter A., Schick F. (2010). Pancreatic fat is negatively associated with insulin secretion in individuals with impaired fasting glucose and/or impaired glucose tolerance: a nuclear magnetic resonance study. Diabetes Metab Res Rev.

[60] Sung K.C., Jeong W.S., Wild S.H., Byrne C.D. (2012). Combined influence of insulin resistance, overweight/obesity, and fatty liver as risk factors for type 2 diabetes. Diabetes Care.

[61] Liu X.X., Liu K.Y., Li P., Han S., Peng X.D., Shen L. (2014). Adiponectin is expressed in the pancreas of high-fat-diet-fed mice and protects pancreatic endothelial function during the development of type 2 diabetes. Diabetes Metab.

[62] Van Meijl L.E.C., Mensink R.P. (2010). Effects of low-fat dairy consumption on markers of low-grade systemic inflammation and endothelial function in overweight and obese subjects: an intervention study. Br J Nutr.

[63] Martins F.F., Marinho T.S., Cardoso L.E.M., Barbosa-da-Silva S., Souza-Mello V., Aguila M.B. (2022). Semaglutide (GLP-1 receptor agonist) stimulates browning on subcutaneous fat adipocytes and mitigates inflammation and endoplasmic reticulum stress in visceral fat adipocytes of obese mice. Cell Biochem Funct.

