# Supplementary material for: Non-alcoholic fatty pancreas disease (NAFPD) as a pre-neoplastic niche: Metabolic and inflammatory Gateways to pancreatic ductal adenocarcinoma
Source: J Clin Transl Endocrinol. 2025 Nov 6;42:100424. doi: 10.1016/j.jcte.2025.100424 (PMC12648718; doi:10.1016/j.jcte.2025.100424)
Supplement: Supplementary Data 1 [file mmc1.docx]

Supplementary files

**Non-Alcoholic Fatty Pancreas Disease (NAFPD) as a Pre-Neoplastic Niche: Metabolic and Inflammatory Gateways to Pancreatic Ductal Adenocarcinoma**

Searched terms;

PubMed 06/14/2025.

("non-alcoholic fatty pancreas disease" OR "pancreatic steatosis") AND ("pancreatic cancer" OR "pancreatic ductal adenocarcinoma") AND ("inflammation" OR "lipotoxicity" OR "metabolic syndrome").

20 results

<https://pubmed.ncbi.nlm.nih.gov/?term=%28%22non-alcoholic+fatty+pancreas+disease%22+OR+%22pancreatic+steatosis%22%29+AND+%28%22pancreatic+cancer%22+OR+%22pancreatic+ductal+adenocarcinoma%22%29+AND+%28%22inflammation%22+OR+%22lipotoxicity%22+OR+%22metabolic+syndrome%22%29>

Scopus

Search within: Article title, Abstracts, Keywords, Authors:

"non-alcoholic fatty pancreas disease" OR "pancreatic steatosis" AND "pancreatic cancer" OR "pancreatic ductal adenocarcinoma" AND "inflammation" OR "lipotoxicity" OR "metabolic syndrome"

Results:

28 documents found

<https://www.scopus.com/results/results.uri?st1=%28plant*+OR+botanical*+OR+phytochemical*%29+AND+%28%CE%B2-lactam*+OR+azetidinone*+OR+%CE%B2-lactone*+OR+aziridine*+OR+epoxyketone*%29+AND+%28Enterobacteriaceae+OR+carbapenem*+OR+KPC+OR+NDM+OR+OXA-48+OR+ESBL%29+AND+%28MIC+OR+antibacterial+OR+%CE%B2-lactamase+OR+inhibitor%29&st2=&s=TITLE-ABS-KEY-AUTH%28%22non-alcoholic+fatty+pancreas+disease%22+OR+%22pancreatic+steatosis%22+AND+%22pancreatic+cancer%22+OR+%22pancreatic+ductal+adenocarcinoma%22+AND+%22inflammation%22+OR+%22lipotoxicity%22+OR+%22metabolic+syndrome%22%29&limit=10&origin=searchbasic&sort=plf-f&src=s&sot=b&sdt=b&sessionSearchId=dcf0f5a6ff27e8eabf0ead35e370d22e>

Limited by document type: Article 14, Review 12. Removed: Book chapter 1, Conference paper 1

Limited by Language type: English 23. Removed: Czech 2, Chinese 1, Hungarian 1, Spanish 1

WOS

Search within Topics:

("non-alcoholic fatty pancreas disease" OR "pancreatic steatosis") AND ("pancreatic cancer" OR "pancreatic ductal adenocarcinoma")

<https://www.webofscience.com/wos/woscc/summary/403329d1-ff67-472a-af95-8a1758cd731f-0168b216be/relevance/1>

45 results

Limited by document type: Article 23, Review Article 19, Removed: Meeting Abstract 2, Editorial Material 1, Proceeding Paper 1

Refined By: Document Types: Review Article or Article. Click to remove this refine from your search. Publication Years: 2015 or 2016 or 2018 or 2019 or 2020 or 2025 or 2024 or 2023 or 2022 or 2021

38 results from Web of Science Core Collection for:

Records removed *before screening*:

Article type: Conference papers (4), Editorial Material (1), and Book chapter (1) were removed.

Non-English Languages: Czech (2), Chinese (1), Hungarian (1), and Spanish (1)

Records identified from Web of PubMed 20,

Scopus 28,

WOS 45,

**Identification**

Records screened

(n =82)

Duplicate records removed 16

**Screening**

Reports sought for retrieval

(n = 66)

Records marked as ineligible by title and abstract screened (n=29)

Studies included in the

review (n=37) and meta-synthesis scientific evidence (n=21)

**Included**

S Figure 1. Flowchart of the PRISMA guideline for the study selection for the meta-synthesis and review

Supplementary Table S1. Risk of Bias assessment

| **References** | **Population (Size / Design)** | **Diagnostic Modality for Pancreatic Fat** | **Event / pathway** | **Key molecules (↑ up; ↓ down)** | **Mechanistic link from steatosis → tumorigenesis** | **Findings Relevant to PDAC** | **Score** |
| --- | --- | --- | --- | --- | --- | --- | --- |
| (Frendi et al., 2024) | * | * | * | * | * | * | 6 |
| (Desai et al., 2020) | * | * | N/A | * | * | * | 5 |
| (Chan et al., 2024) | * | * | N/A | N/A | * | * | 4 |
| (Chen et al., 2017) | * | N/A | * | * | * | * | 5 |
| (Fukuda et al., 2011) | * | * | * | * | * | * | 6 |
| (Fukuda et al., 2025) | * | * | * | * | * | * | 6 |
| (Fukuda et al., 2017) | * | * | * | N/A | * | * | 5 |
| (Fukui et al., 2019) | * | * | * | N/A | * | * | 5 |
| (Hoogenboom et al., 2021) | * | * | * | N/A | * | * | 5 |
| (Lesmana et al., 2018) | * | * | * | N/A | * | * | 5 |
| (Liu et al., 2022) | * | N/A | * | * | * | * | 5 |
| (Mathur et al., 2009) | * | * | * | N/A | * | * | 5 |
| (Philip et al., 2013) | * | N/A | * | * | * | * | 5 |
| (Tang et al., 2017) | * | N/A | * | * | * | * | 5 |
| (Toste et al., 2015) | * | N/A | * | * | N/A | * | 4 |
| (Van Audenaerde et al., 2017) | * | N/A | * | * | N/A | * | 4 |
| (Vlăduț et al., 2025) | * | * | * | * | N/A | * | 5 |
| (Wang et al., 2023) | * | N/A | * | * | * | * | 5 |
| (Yamazaki et al., 2024) | * | * | * | N/A | * | * | 5 |
| (Zhang et al., 2023) | * | N/A | * | * | N/A | * | 4 |
| (Zhou et al., 2021) | * | * | * | N/A | * | * | 5 |
